# The Complete Mitochondrial Genome of *Gossypium hirsutum* and Evolutionary Analysis of Higher Plant Mitochondrial Genomes

**DOI:** 10.1371/journal.pone.0069476

**Published:** 2013-08-05

**Authors:** Guozheng Liu, Dandan Cao, Shuangshuang Li, Aiguo Su, Jianing Geng, Corrinne E. Grover, Songnian Hu, Jinping Hua

**Affiliations:** 1 Department of Plant Genetics and Breeding, College of Agronomy and Biotechnology, Key Laboratory of Crop Heterosis and Utilization of Ministry of Education, Beijing Key Laboratory of Crop Genetic Improvement, China Agricultural University, Beijing, China; 2 CAS Key Laboratory of Genome Sciences and Information, Beijing Institute of Genomics, Chinese Academy of Sciences, Beijing, China; 3 Department of Ecology, Evolution, and Organismal Biology, Iowa State University, Ames, Iowa, United States of America; University of Georgia, United States of America

## Abstract

**Background:**

Mitochondria are the main manufacturers of cellular ATP in eukaryotes. The plant mitochondrial genome contains large number of foreign DNA and repeated sequences undergone frequently intramolecular recombination. Upland Cotton (*Gossypium hirsutum* L.) is one of the main natural fiber crops and also an important oil-producing plant in the world. Sequencing of the cotton mitochondrial (mt) genome could be helpful for the evolution research of plant mt genomes.

**Methodology/Principal Findings:**

We utilized 454 technology for sequencing and combined with Fosmid library of the *Gossypium hirsutum* mt genome screening and positive clones sequencing and conducted a series of evolutionary analysis on *Cycas taitungensis* and 24 angiosperms mt genomes. After data assembling and contigs joining, the complete mitochondrial genome sequence of *G. hirsutum* was obtained. The completed *G.hirsutum* mt genome is 621,884 bp in length, and contained 68 genes, including 35 protein genes, four rRNA genes and 29 tRNA genes. Five gene clusters are found conserved in all plant mt genomes; one and four clusters are specifically conserved in monocots and dicots, respectively. Homologous sequences are distributed along the plant mt genomes and species closely related share the most homologous sequences. For species that have both mt and chloroplast genome sequences available, we checked the location of cp-like migration and found several fragments closely linked with mitochondrial genes.

**Conclusion:**

The *G. hirsutum* mt genome possesses most of the common characters of higher plant mt genomes. The existence of syntenic gene clusters, as well as the conservation of some intergenic sequences and genic content among the plant mt genomes suggest that evolution of mt genomes is consistent with plant taxonomy but independent among different species.

## Introduction

Mitochondria, where the oxidative phosphorylation and the various biochemical processes take place throughout metabolism, are the main manufacturers of cellular ATP in eukaryotes. The plant mitochondrial genome contains large number of foreign DNA and repeated sequences undergone frequently intramolecular recombination, making it extraordinarily difficult to sequence plant mitochondrial (mt) genomes, particularly those of angiosperms [1], [2]. With the sequencing efforts over the past decade, the number of complete mt genomes has been greatly increased [3]–[5]. These finished mt genomes allow a deep analysis on the evolution of the higher plant mt genomes in aspects of gene orders, genome structure, and migration sequences as well as phylogenetic analysis.

Angiosperm mt genomes vary dramatically in size [6]. The size variation likely stems from their tendency to integrate DNA from other genomes [7]–[11] and the propensity for repeated sequences [12], [13]. Even so, large numbers of homologous sequences are distributed through the plant mt genome, including many noncoding sequences. Compared the mt genome of *Brassica napus* with that of *Arabidopsis thaliana* and *Beta vulgaris*, the values of shared sequences were in good agreement with the phylogenetic relationship among these three species [14].

Because of low rates of nucleotide substitution [15], [16], the mitochondrial genes are often used in plant evolutionary analysis, especially for construction of ancient phylogenetic relationships [5], [17], [18]. MtDNA trees are largely congruent with those constructed with chloroplast genes and nuclear genes, showing that mt genes are informative markers for evolution analysis across angiosperms. Gene orders are frequently not conserved across species [16], [19], possibly due to the mitochondrial penchant for recombination [12], [20]. Conservation of gene clusters are frequently used to infer evolution relationship among animal mt genomes [21], however, little researches have been performed in plant mt genomes [22], [23].

Here we report the first complete *Gossypium* mt genome derived from the widely cultivated upland species, *Gossypium hirsutum*. This sequence represents a major circular molecule that is 621,884 bp in length. The upland cotton mt genome possesses most of the common characters of higher plant mt genomes and maintains essential protein-coding genes and tRNA genes. Phylogenetic analyses, as well as analyses of conserved sequences, tRNAs and gene clusters among 25 mt genomes (24 angiosperms and *Cycas taitungensis*), indicate that (1) evolution of mt genomes is independent among different species, and (2) the evolution of the mt genomes is consistent with plant taxonomy as a whole (the upland cotton mt genome is much closer with *Carica papaya* than other angiosperms).

## Results and Discussion

### Genome assembly and features of *Gossypium hirsutum* mitochondrial genome

#### 1. Genome assembly

The *Gossypium hirsutum* mt genome was sequenced using the Roche 454 GS FLX platform, which generated 286,792 reads with an average length of 399 bp. Cleaned reads were assembled by Newbler (Version 2.53), and contigs were subsequently joined via PCR into three scaffolds according to the from-to relationship among contigs (Table S1). Primers were designed and used to screen a Fosmid library [24] for clones to join the three scaffolds. Of the eight identified positive clones, two clones were selected for shotgun sequencing to finish the gaps, while end-sequencing of the remaining six clones were performed to verify the finished genome. Finally, the upland cotton mt genome was assembled into a single, circular molecule, with the length 621,884 bp and GC content 45.0% (Accession Number JX065074).

#### 2. Gene annotation

**Figure 1:**
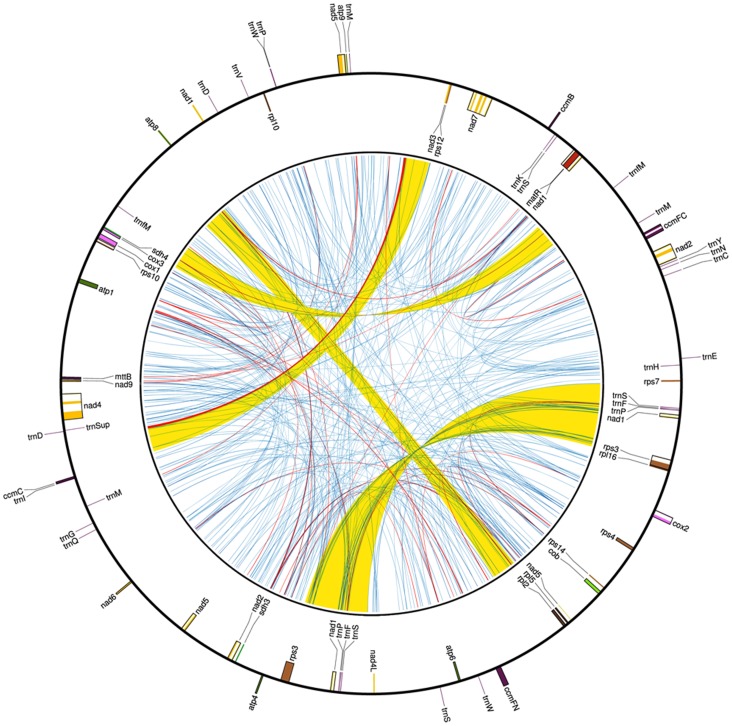
Genome map of *Gossypium hirsutum* mitochondrial genome. The map shows both the gene map (outer circle) and repeat map (inner map). Genes exhibited on the inside of outer circle are transcribed in a clockwise direction, while genes on the outside of outer circle are transcribed in a reverse direction. The inner circle reveals the distribution of repeats in *G. hirsutum* mt genome. The yellow lines represent > = 1 kb repeats, the blue lines represent <100 bp repeat and the red lines represent repeat between 100 bp and 1 kb.

68 genes were annotated in the cotton mt genome, including 35 protein-coding genes, four rRNA genes and 29 tRNA genes (Figure 1, Table S2). Among the eight multi-copy genes (i.e., *nad1*, *rps3*, *rrn26*, *trnW*, *trnS*(GCT), *trnP*, *trnfM* and *trnM*), *nad1* gene contains an additional copy with exon b and exon c, and *rps3* gene has an extra pseudogene-like copy which lacks 544 bp on the 3′ end of exon 2. Five genes (*rps1*, *rps2*, *rps11*, *rps13* and *rps19*) are partially deleted and several remnant fragments of those deleted loci are annotated in the genome, with the largest fragment only 54 bp in length (derived from *rps19*). The 1.5 kb intron of *rpl2* gene reported in other sequenced higher plant mt genomes is not found in the *G. hirsutum* mt genome.

**Figure 2:**
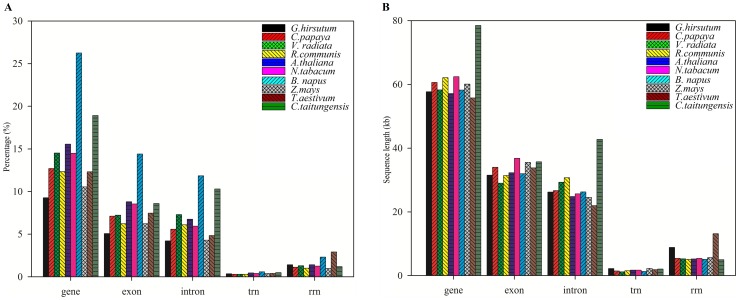
Gene composition of different mitochondrial genomes. The percentage of different genic sequence (A) and the length of different genic sequence (B).

The protein-coding genes in the upland cotton mt genome comprise a total length of 61,582 bp (9.9%), nearly half of which is intronic sequence (exons  = 31,721 bp; introns  = 29,861 bp), while tRNA genes and rRNA genes only represent 2,234 bp and 8,826 bp of the genome. The percentages of genic contents except the tRNA content differ significantly due to the variation of mt genome size in angiosperms (Figure 2A). However, the sequence length distribution is very similar to other sequenced seed plant mt genomes, with the exception of the rRNA content (Figure 2B); it is slightly elevated in the *G. hirsutum* mt genome due to the duplication of *rrn26* (3,374 bp).

#### 3. Gene clusters

**Figure 3:**
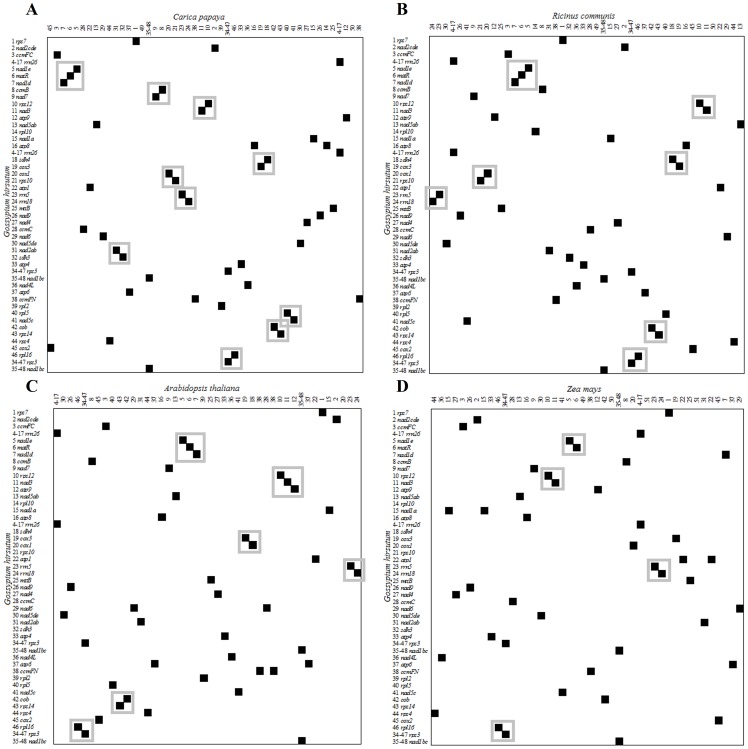
Gene order and existed clusters between the mitochondrial gene maps of *Gossypium* and other four angiosperms. Gene order of the protein-coding and rRNA-coding genes, and the former's trans-spliced exons were based on the mt genome of *G. hirsutum* arranging from top to bottom. Genes of other four mt genomes were indicated by the corresponding numbers given to cotton genes listed on the left margin. Duplicate genes carried the same number. From left to right for (A) *C. papaya*, (B) *R. communis*, (C) *A. thaliana* and (D) *Z. mays*.

Except the tRNA genes, ten gene clusters are annotated in the upland cotton mt genome (Table 1). Genes that comprise such clusters are usually separated by short intergenic regions or even partially overlapped in coding sequences and transcribed from the same strand. The gene orders differ markedly in higher plant mt genomes and four plant mt genomes are chosen to compare the gene orders with *G. hirsutum* mt genome. As showed in Figure 3, the *G. hirsutum* mt genome shares 10 clusters with *C. papaya* (Figure 3A), seven with *R. communis* (Figure 3B), six and four with *A. thaliana* (Figure 3C) and *Z. mays* (Figure 3D).

**Table 1:** Information of gene clusters in Gossypium hirsutum mt genome.

Gene cluster	Location and Interval	Type
***rpl16-rps3***	593447..593881**-(-28bp**)-593853..597250	III
***cob-rps14***	547956..549134-(**1363bp**)-550498..550800	II
***rpl2-rpl5-nad5c***	532722..533726-(**497bp**)-534224..534805-(**1117bp**)-535923..535944	II
***nad2abc-sdh3***	418598..420365-(**999bp**)-421265..421699	I
***mttB-nad9***	308702..309502-(**184bp**)-309687..310259	IV
***sdh4-cox3***	258268..258666**-(-72bp**)-258594..259391	I
***cox1-rps10***	260808..262400-(**186bp**)-262587..263768	II
***atp9-nad5ab***	162829..163140-(**220bp**)-163361..165659	I
***nad3-rps12***	129383..129754-(**48bp**)-129803..130159	II
***nad1e-matR-nad1d***	81154..81412-(**806bp**)-82219..84186-(**661bp**)-84848..84905	IV

**Boldface**: Interval length between two genes.

Type I represents gene cluster composed of respiratory genes; Type II represents gene cluster composed of respiratory genes; Type III represents gene cluster composed of respiratory genes; Type IV represents gene cluster compose of respiratory genes.

#### 4. Repeated sequence

343 repeat sequences larger than 20 bp were detected in *G. hirsutum* mt genome (Figure 1). In total, the detected repeats occupied 22.9% of the mt genome. Of the 343 repeats, most of them exist as short (20 bp to 39 bp), scattered repeats, about 10% (35 repeats) are larger than 100 bp (Table 2) and 1% (four repeats) larger than 10 kb, (R1, 27,495 bp; R2, 10,623 bp; R3 10,302 bp; and R4, 10,251 bp). Copy number for the larger repeats (100+ bp) varied narrowly from two (22 repeats) to four (two repeats) copies. The smaller repeats were also tabulated, and appeared to have distinct distributions and copy number variations (Table 3).

**Table 2:** Repeats (>100 bp) in *Gossypium hirsutum* mt genome.

No.	Size (bp)	Identity (%)	Copy-1		Copy-2^a^		Copy-3^a^		Copy-4^a^		Type^b^
			start	end	start	end	start	end	start	End	
R01	27495	99.92	437002	464489	594397	621884					DR
R02	10623	99.81	224949	235564	**532802**	**522190**					IR
R03	10302	99.98	130185	140486	**340674**	**330373**					IR
R04	10251	99.86	64505	74747	247693	257941					DR
R05	879	100	140497	141375	**330372**	**329494**					IR
R06	399	99.5	225720	226118	**421200**	**420802**	**532032**	**531634**			IR/DR
R07	349	99.43	81010	81358	**226466**	**226118**	531286	531634			IR/DR
R08	260	86.54	519189	519438	**555385**	**555140**					IR
R09	259	98.07	226519	226775	**273990**	**273734**	**531233**	**530978**			IR/DR
R10	256	83.2	56397	56644	**68151**	**67904**	**251342**	**251095**			IR/DR
R11	229	99.13	39574	39802	**119894**	**119666**					IR
R12	203	99.51	260466	260668	**430908**	**430706**					IR
R13	194	100	70998	71191	254191	254384	427256	427449			DR
R14	175	100	147589	147763	495709	495883					DR
R15	174	98.85	455621	455794	550847	551020	613016	613189			DR
R16	168	91.07	378953	379120	**536243**	**536080**					IR
R17	166	94.58	162194	162357	**310412**	**310248**					IR
R18	162	91.98	278372	278532	**506946**	**506786**					IR
R19	160	97.5	225357	225515	**439973**	**439816**	**532394**	**532236**	**597366**	**597208**	IR/DR
R20	159	99.37	278884	279042	455305	455463	612700	612858			DR
R21	151	100	427472	427622	**486639**	**486489**					IR
R22	145	87.59	246993	247137	**409294**	**409158**					IR
R23	138	97.1	260510	260645	**430864**	**430729**	495627	495763			IR/DR
R24	136	96.32	285066	285201	**534095**	**533960**					IR
R25	135	90.37	519189	519320	**555385**	**555255**					IR
R26	133	94.74	161921	162053	**351301**	**351169**					IR
R27	128	84.38	278561	278686	**506757**	**506635**					IR
R28	127	92.91	279043	279168	455473	455598	612868	612993			DR
R29	123	88.62	260519	260638	**430855**	**430736**	495636	495755	**578212**	**578092**	IR/DR
R30	118	99.15	185254	185371	**309736**	**309619**					IR
R31	113	100	81796	81908	**438852**	**438740**	**596247**	**596135**			IR/DR
R32	113	98.23	86305	86417	**307767**	**307655**					IR
R33	107	100	70936	71042	254129	254235	**502859**	**502753**			IR/DR
R34	101	85.15	302711	302803	513954	514053					DR
R35	101	97.03	425155	425255	**555258**	**555158**					IR

a Boldface: IR copy, compared with copy-1 as control.

b DR and IR: direct and reverse repeats, respectively; IR/DR: both direct repeat and reverse repeat among multiple copies.

**Table 3:** Frequency distribution of repeat lengths in the mt genome of *Gossypium hirsutum.*

Size, bp	20–39	40–59	60–79	80–99	100–999	> = 1000
Number	192	69	35	11	32	4
Total length of repeats, bp	10, 747	9, 667	9, 567	8, 365	18, 368	117, 300
Coverage, %	1.7	1.6	1.5	1.3	3.0	18.9

#### 5. Cp-like sequences

Integration of cp-like sequences is a common occurrence in plant mitochondrial genomes, and *G. hirsutum* is no exception. 27 chloroplast-derived sequences (80% or higher identity to the *G. hirsutum* chloroplast genome) are found in the mt genome, contributing 6,833 bp (1.1% of the genome size) with segments ranging from 36 bp to 2,185 bp. 12 of the 27 chloroplast-derived sequences are tRNA related sequences, three are photosynthesis related sequences and the rest are other type of chloroplast sequences.

### Migration of cpDNA in plant mt genomes

Chloroplast-derived sequences play an important role in plant mt genomes. Many researches have shown that cp-like tRNA genes are essential to maintain normal translation [18], [25]–[27] and cp-like sequences can act as functional genes and gene promoters [28], [29]. Besides, mitochondrial plastid DNA also contributes codons to mitochondrial protein-coding sequences and has a role in posttranscriptional RNA processing [10].

14 species that chloroplast genomes are available were chosen to analyze cp-like migration in plant mt genomes (Table 4). The length of individually integrated sequences varies widely, from 20 bp to 12 kb. The capacity of cpDNA in plant mt genomes also differs greatly; the total amount of cpDNA exceeds 60 kb in *Vitis vinifera* mt genome, whereas it represents less than 2 kb in *Silene latifolia* and *Vigna radiate*. Besides, the size of the largest integrated fragment varied from 275 bp (*Silene latifolia*) to 12 kb (*Carica papaya*). Based on the above data, the migration of cpDNA in plant mt genomes seems to be an independent and random event.

**Table 4:** Information of chloroplast homologous sequences in plants.

Species	Total length of chloroplast homologous sequence in mt genome	Numbers of chloroplast homologs	Coverage of chloroplast homologous sequence
*Arabidopsis thaliana*	4803	24	1.3%
*Brassica napus*	8749	23	3.9%
*Carica papaya*	21368	25	4.5%
*Nicotiana tabacum*	11184	37	2.6%
*Cucurbita pepo*	88208	204	9.0%
*Gossypium hirsutum*	6833	27	1.1%
*Vigna radiata*	2109	17	0.5%
*Vitis vinifera*	64357	73	8.3%
*Ricinus communis*	5649	26	1.1%
*Silene latifolia*	1998	16	0.8%
*Sorghum bicolor*	26357	45	5.6%
*Triticum aestivum*	13855	36	3.1%
*Zea mays*	23445	39	4.1%
*Oryza sativa ssp indica*	33176	41	6.7%
*Oryza sativa ssp japonica*	33157	41	6.7%

**Figure 4:**
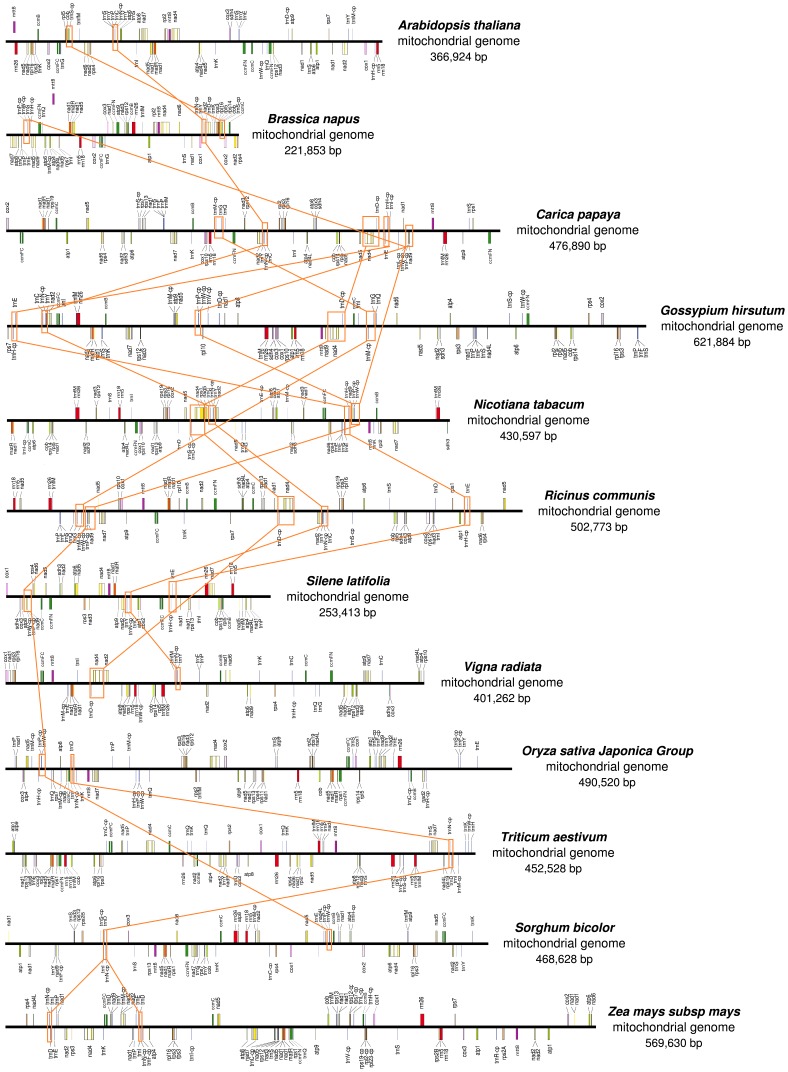
Linkage map between cp-homologous and mitochondrial sequences in higher plant mt genomes.

Blast was performed to check the homology of cp-like migration in plant mt genomes. Five cp-derived fragments (*trnH*, *trnM*, *trnN*, *trnP* and *trnW*) were found conserved in all analyzed mt genomes and one (*trnD*) and two (*trnC* and *trnF*) cp-derived fragment were found conserved in dicots and monocots, respectively. In addition, some of these conserved cp-derived fragments maintain the same sequence arrangement relationship with mitochondrial genes (Figure 4), indicating these migration events are very ancient and occurred before the species differentiation.

### Origin and distribution of tRNAs in plant mt genomes

The ancestral mt genome possesses an intact set of transfer RNAs (tRNAs), however, a large number of tRNAs undergo loss, migration and inactivation during mt genome evolution [27]. Different with the human mt genome, which keeps a minimal but complete set of tRNA genes, the number of tRNA genes in numerous plant mt genomes is insufficient for translation, although a certain number of tRNA genes have been brought in via DNA migration [30]–[32].

To evaluate the origin and distribution of tRNA genes, tRNAscan-SE [33] was adopted to predict the number and types of tRNA genes. Most of the analyzed species keep 16–21 kinds of tRNA genes and because of extensive loss of genes in *Silene latifolia* [18], only 10 were annotated. These results suggest that nuclear encoded tRNAs are necessary to maintain the normal translation in higher plant mt genomes.

**Figure 5:**
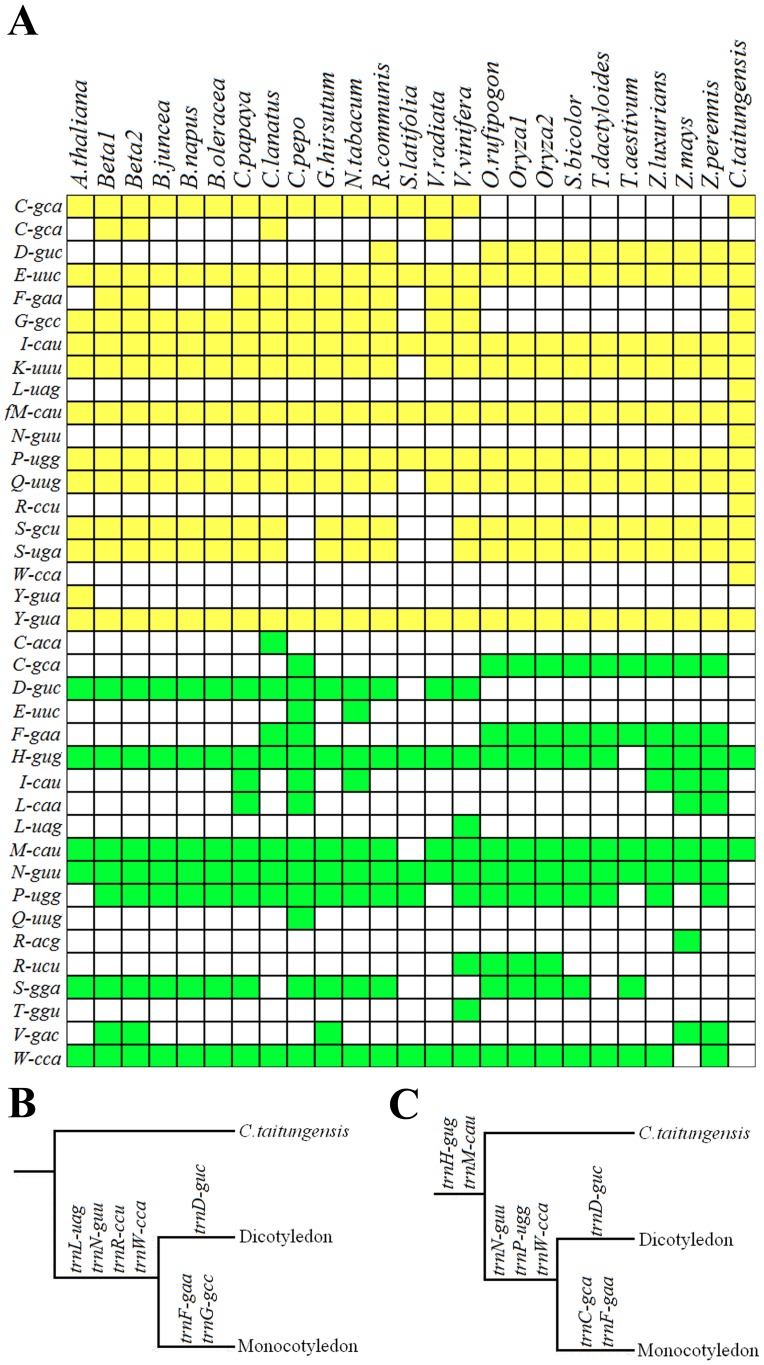
Distribution map of tRNA genes in 25 plants. Figure 5A shows distribution of tRNA genes in higher plant mt genomes: the yellow boxs represented native tRNA genes, the green cells represent cp-like tRNA genes; Figure B shows uptake of cp-like tRNA genes during different evolutionary period; Figure C shows loss of native tRNA genes during different evolutionary period. The three *Oryza* genomes: 1, *Oryza rufipogon*; 2, *Oryza sativa subsp indica*; 3, *Oryza sativa subsp japonica*. The two *Beta* genomes are: 1, *Beta vulgaris subsp maritima*; 2, *Beta vulgaris subsp vulgaris*.

Based on chloroplast genomes, 19 native (mitochondria-originated) tRNA genes and 19 cp-like tRNA genes are defined in higher plant mt genomes (Figure 5A). As showed in Figure 5B and 5C, although we found the uptake of four cp-like tRNAs (*trnD*, *trnF*, *trnN*, and *trnW*) and lose of four native tRNAs (*trnD*, *trnF*, *trnN*, and *trnW*) seems to occur during the same period, the uptake and lose of the rest cp-like tRNA genes (Figure 5B) and native tRNA genes (Figure 5C) are more likely to be occurred in different period of evolution. Besides, some cp-like tRNA genes have scattered distribution and some native tRNA genes are irregularly lost among higher plant mt genomes, showing the gain and lose of tRNA genes occurred independently during the evolution.

### Gene orders and gene clusters in plant mt genomes

The gene orders differ tremendously among plant mt genomes. In this research, we compared the gene orders across the 25 species and counted the number of syntenic gene clusters (genes that keep the same order; Table 5). In general, the closer species in evolution share more clusters. However, there are also some inconsistent cases, *C. taitungensis* and *C*. *lanatus* share 13 gene clusters, much more than the number between *C*. *lanatus* and the other angiosperms; the cluster number between *T*. *aestivum* and *C*. *lanatus* is larger than that between *C*. *lanatus* and the other dicots. These exceptions probably were due to the frequently recombination during the plant mt genomes. Recombination can break the previous clusters and result in novel ones, while multiple recombination events can lead to generate the same synteny gene clusters too.

**Table 5:** Numbers of synteny gene clusters across 25 plant mt genomes.

Species	Ct	At	Bvm	Bvv	Bj	Bn	Bo	Cap	Cl	Cup	Gh	Nt	Rc	Sl	Vr	Vv	Or	Ori	Orj	Sb	Td	Ta	Zl	Zm
*A. thaliana*	8																							
*Beta1*	8	6																						
*Beta2*	7	6	39																					
*B. juncea*	8	19	6	7																				
*B. napus*	8	19	6	7	39																			
*B. oleracea*	8	19	6	7	39	39																		
*C. papaya*	11	11	8	8	11	11	11																	
*C. lanatus*	13	9	10	10	10	10	11	16																
*C. pepo*	13	10	9	10	9	9	10	16	23															
*G. hirsutum*	9	8	4	4	9	9	9	11	12	12														
*N. tabacum*	10	8	9	9	8	8	8	14	14	16	11													
*R.communis*	12	10	11	11	10	9	10	14	18	16	8	15												
*S. latifolia*	7	7	10	10	7	7	7	10	8	9	6	12	9											
*V. radiata*	10	10	6	6	11	10	11	11	11	11	10	12	13	8										
*V. vinifera*	12	10	10	10	12	12	12	12	17	14	7	12	13	9	10									
*O. rufipogon*	9	6	5	5	5	6	6	7	10	9	6	6	7	5	6	10								
*Oryza1*	9	5	5	5	5	6	6	7	10	9	6	5	7	5	6	10	40							
*Oryza2*	9	5	5	5	5	6	6	7	10	9	6	5	7	5	6	10	40							
*S. bicolor*	7	4	6	5	5	4	4	6	8	5	5	5	5	2	5	9	8	9	9					
*T. dactyloides*	6	6	4	4	6	5	5	7	6	5	6	5	7	2	5	6	8	9	9	7				
*T. aestivum*	7	6	5	5	8	6	6	10	11	10	6	8	9	4	7	8	11	11	11	9	10			
*Z. luxurians*	7	7	5	5	5	5	5	7	8	6	5	6	6	3	5	7	9	9	9	11	17	11		
*Z. mays*	6	6	6	6	6	6	6	9	8	7	4	5	6	4	5	8	11	9	9	10	13	11	20	
*Z. perennis*	7	8	6	6	6	7	7	7	9	7	4	8	6	3	5	8	10	11	11	11	15	11	30	17

Note: Numbers of synteny gene clusters differed across *C. taitungensis* (Ct), *A. thaliana* (At), *Beta vulgaris subsp maritima* (Bvm), *Beta vulgaris subsp vulgaris* (Bvv), *B. juncea* (Bj), *B. napus* (Bn), *B. oleracea* (Bo), *C. papaya* (Cap), *C. lanatus* (Cl), *C. pepo* (Cup), *G. hirsutum* (Gh), *N. tabacum* (Nt), *R.communis* (Rc), *S. latifolia* (Sl), *V. radiata* (Vr), *V. vinifera* (Vv), *O. rufipogon* (Or), *Oryza sativa subsp indica* (Ori), *Oryza sativa subsp japonica* (Orj), *S. bicolor* (Sb), *T. dactyloides* (Td), *T. aestivum* (Ta), *Z. luxurians* (Zl), *Z. mays* (Zm) and *Z. perennis*. The two Beta genomes in the first row were: 1, *Beta vulgaris subsp maritima*; 2, *Beta vulgaris subsp vulgaris*, and the two Oryza genomes in the first row were: 1, *Oryza sativa subsp indica*; 2, *Oryza sativa subsp japonica*. Any two genes linked were counted as one synteny gene cluster.

**Figure 6:**
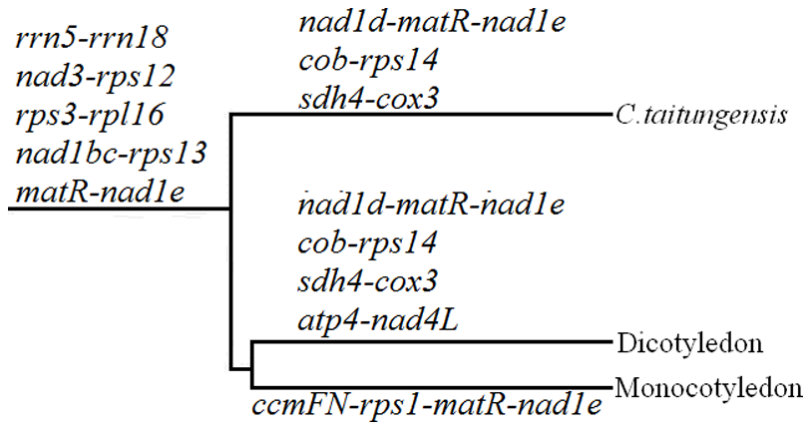
Distribution of conserved gene clusters.

There are also some conserved syntenic gene clusters among higher plant mt genomes. Alverson reported that 14 syntenic gene clusters are shared between *C*. *lanatus* and *C. pepo* [13]. We checked gene clusters in the 25 mt genomes and found five gene clusters conserved in all the plant mt genomes (Figure 6 and Table 6). There are also four and one gene clusters that are specific conserved in dicots and monocots respectively. The genes that compose these clusters share short intergenic region or even overlap in the CDS region. The gene cluster *atp4*-*nad4L*, for example, exists in all dicots surveyed, except for *Gossypium hirsutum*; the cluster *nad1e-matR* exists in all the plant but *Beta*, *Nicotiana* and *Silene*, indicating a lineage specific disruption of this cluster.

**Table 6:** Distribution of closely linked clusters in *Gossypium hirsutum* and other plant mt genomes.

	*rrn5-* *rrn18*	*nad3-* *rps12*	*rps3-* *rpl16*	*nad1d-* *matR-* *nad1e*	*nad1d-* *matR*	*matR-* *nad1e*	*sdh4-* *cox3*	*cob-* *rps14*	*nad1bc-* *rps13*	*atp4-* *nad4L*	*ccmFn-* *rps1-matR-* *nad1e*	*ccmFN-* *rps1*
*C.taitungensis*	+	+	+	+	+	+	+	+	+	−	−	+
*A.thaliana*	+	+	+	+	+	+	+	+	#	+	#	#
*Beta1*	+	+	#	−	+	−	+	#	+	+	−	#
*Beta2*	+	+	#	−	+	−	+	#	+	+	−	#
*B.juncea*	+	+	+	+	+	+	+	+	#	+	#	#
*B.napus*	+	+	+	+	+	+	+	+	#	+	#	#
*B.oleracea*	+	+	+	+	+	+	+	+	#	+	#	#
*C.papaya*	+	+	+	+	+	+	+	+	+	+	−	+
*C.lanatus*	+	+	+	+	+	+	+	+	+	+	−	+
*C.pepo*	+	+	+	+	+	+	+	+	+	+	−	−
*G.hirsutum*	+	+	+	+	+	+	+	+	#	−	#	#
*N.tabacum*	+	+	+	−	+	−	+	+	+	+	−	−
*R.communis*	+	+	+	+	+	+	+	+	+	+	−	−
*S. latifolia*	+	#	#	−	+	−	#	+	+	+	−	#
*V. radiata*	+	+	+	+	+	+	+	+	#	+	−	−
*V. vinifera*	+	+	+	+	+	+	+	+	+	+	−	−
*O.rufipogon*	+	+	+	−	−	+	#	−	+	−	+	+
*Oryza1*	+	+	+	−	−	+	#	−	+	−	+	+
*Oryza2*	+	+	+	−	−	+	#	−	+	−	+	+
*S.bicolor*	+	+	+	−	−	+	#	−	+	−	+	+
*T.dactyloides*	+	+	+	−	−	+	#	#	+	−	+	+
*T.aestivum*	+	+	+	−	−	+	#	−	+	−	+	+
*Z.luxurians*	+	+	+	−	−	+	#	#	+	−	+	+
*Z.mays*	+	+	+	−	−	+	#	#	+	−	+	+
*Z.perennis*	+	+	+	−	−	+	#	#	+	−	+	+

**Note:**

**+**, presence of the gene cluster; −, absence of the gene cluster; **#**, absence for gene lose.

The two *Oryza* genomes are: 1, *Oryza sativa* Indica Group; 2, *Oryza sativa* Japonica Group.

The two *Beta* genomes are: 1, *Beta vulgaris subsp. maritima*; 2, *Beta vulgaris subsp. vulgaris*.

The origin of conserved syntenic gene clusters is still unclear. According to the *Ka*/*Ks* ratio of these gene clusters, most of them undergo purify selection and the remaining undergo neutral evolution, indicating the important role of natural selection on these gene clusters. The genes in each of gene clusters are transcribed from the same strand, implying that they may function in a co-transcription manner; the *rps3*-*rp116*-*nad3*-*rps12* cluster in rice shares the same promoter and undergoes co-transcription [34]; three clusters (*rrn5-rrn18*, *rps3-rpl16* and *nad3-rps12*) were reported co-transcribed in *Phoenix dactylifera* [35]. Besides, these clusters may also be helpful to predict functional coupling between genes in angiosperms [36].

### Conserved sequence and phylogenetic analysis

Homologous sequences are distributed among the plant mt genomes, including a certain region of non-coding sequences. To calculate the length of shared sequences among different species, the chloroplast-derived sequences and extra copies of large repeats were removed from the analyzed mt genomes before blasting against the other mt genomes. As shown in Table S3, species closely related share the most sequences, even outside of the coding regions; species belong to different families share fewer and species belong to different groups (gymnosperm, monocots and dicots) share the fewest. These results indicate that the length of homologous sequence among plant mt genomes is consistent with taxonomy, despite the exceptional variability among these mt genomes. The *Silene latifolia*, member of the Caryophyllaceae family, is the least-shared species among the 24 angiosperms because of extensive loss of genomic sequence [18].

**Figure 7:**
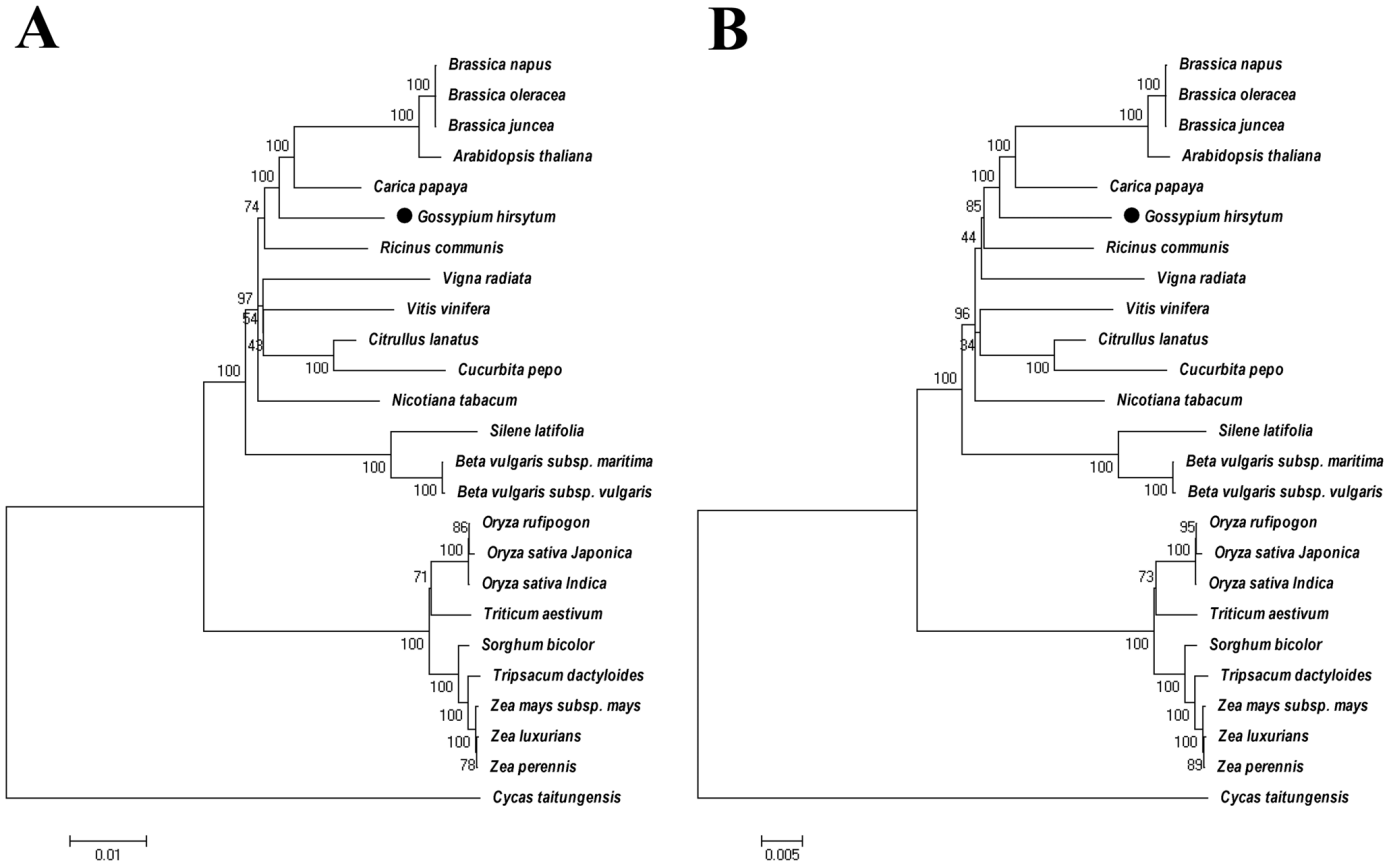
Phylogenetic trees of 21 respiratory related genes. The ML tree (A) and the NJ tree (B). Genes used were listed in Table S4, including 17 respiratory complex genes and four cytochrome c biogenesis genes.

**Figure 8:**
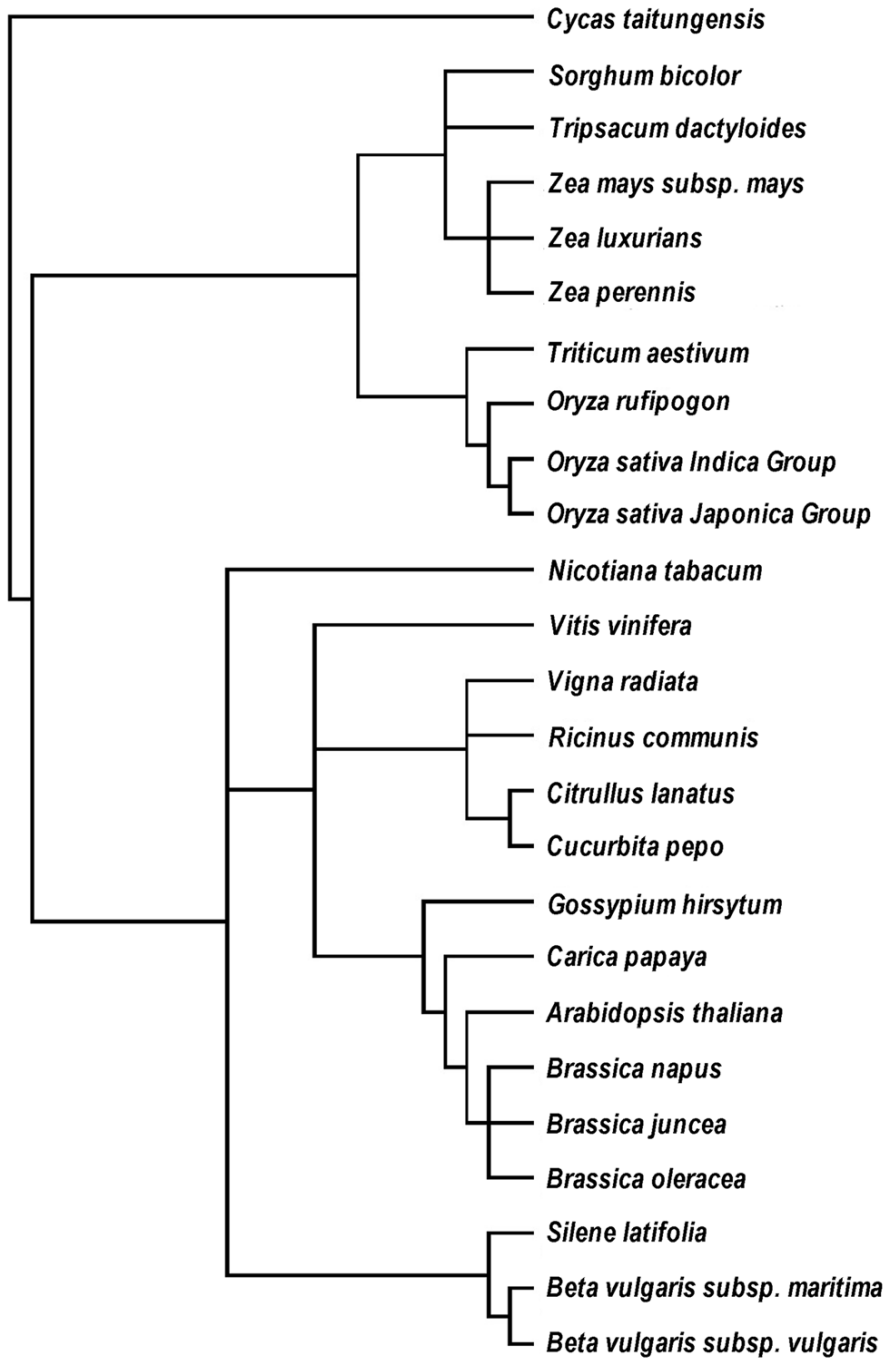
NCBI common tree of 25 analyzed species.

21 respiratory chain related genes that exist in all higher plants were selected for phylogenetic analysis (Table S4), including 17 respiratory complex genes and four cytochrome c biogenesis genes. These genes were first concatenated in a head-to-tail format, and phylogenetic trees were completed with both maximum likelihood method (ML; Figure 7A) and neighbor-joining (NJ; Figure 7B) method. The phylogenetic trees were congruent with the plant taxonomy and NCBI taxonomy common tree (Figure 8). To further assess the utility of the mt genes in phylogenetic reconstruction, these 21 were divided into five groups according to the function of their proteins, and genes in each group were assembled in a head-to-tail arrangement. These trees show more or less differences with the common tree. Three of the five functional groups (Complex I, V and cytochrome c biogenesis genes) reconstruct the divergence of monocots and dicots but showing slightly different evolution relationships (Figure S1 and Figure S2), the Complex III and IV gene sets fail even to reconstruct the monocot-dicot division (Figure S3).

The evolutionary rate of mitochondrial genes varies greatly among plant species [5], phylogenetic analysis of single gene differs with the plant taxonomy. In this research, we tried phylogenetic analysis of functional groups and 21 conserved genes. Compared with previous reports [5], [17], [18], phylogenetic tree of 21 conserved mitochondrial genes shows best coincidence with NCBI taxonomy common tree.

## Conclusion

Plant mitochondrial genomes are fascinating molecules, whose lability and striking differences in evolutionary rates among genic and intergenic regions have generated significant interest. The *G. hirsutum* mt genome possesses most of the common characters of higher plant mt genomes. The comparative analysis presented here allows a more comprehensive understanding of mitochondrial genome evolution in higher plant. The existence and conservation of gene clusters, origin and distribution of tRNA genes, as well as the conservation of some intergenic sequences and genic contents suggest that evolution of mt genomes is consistent with plant taxonomy. But the highly dynamic genome structures (genome size, gene orders and gene content) reflects that recombination of higher plant mt molecular is independent and random among different species.

## Materials and Methods

### Plant material and mitochondrial DNA extraction

Mitochondria were obtained from 7-days-old etiolated seedlings of a variety of upland cotton (*Gossypium hirsutum* L.), ‘Sumian No. 20 (Xu244)’. Etiolated seedlings were ground with homogenate buffer in the proper proportion and after pulping, nuclei and debris were removed by centrifugation at 3,000 rpm for 16 min at 4°C, the supernatant was transferred to a new tube and centrifugation was carried out for 40 min at 8,500 rpm at 4°C to isolate mitochondria. Purified mitochondria were obtained by discontinuous sucrose density gradient centrifugation. After digestion of nuclear DNA with DNase I, mitochondria were lysed by CTAB at 65°C for 30 min. The lysis solution was extracted by chloroform: isoamyl alcohol for 2–3 times and then absolute ethyl alcohol was used to precipitate the mitochondrial DNA (mtDNA).

### Genome sequencing and assembly

Upland cotton mtDNA were sequenced using 454 in Beijing Institute of Genomics, Chinese Academy of Sciences. Purified mtDNA was used to construct sequencing library, according to the manufacturer's manual for the 454 GS FLX Titanium. The reads were assembled into contigs by 454 GS FLX platform after removing the adaptor and contaminant sequences [37].

The relationship among contigs was acquired according to the from-to relationship. Then, primers were designed to join the contigs and fill the genomic gaps. After sequencing of PCR bands, the contig were assembled in scaffolds.

### Mitochondrial genome library construction and clone sequencing

Mitochondrial genome Fosmid library for *G. hirsutum* was constructed following CopyControl Fosmid Library Production Kit (Epicentre, Cat. No. CCFOS110). Mitochondria genomic DNA was random mechanical sheared, size-fractioned by pulsed field gel electrophoresis, and ligated to pCC1FOS vector. The packaged phage infected the EpI300-T1^R^ host cell and then well-separated colonies were randomly picked to accomplish the fosmid library construction [24].

The library was screened by primers designed on the conserved genes and scaffold terminals. The positive clones were chose for shotgun sequencing in Beijing Institute of Genomics, Chinese Academy of Sciences. The terminal sequencing of positive clones operated in Invitrogen Life Technologies Corporation.

### Genome annotation and sequence analysis

Just like the method described in Alverson's report [13], a local database was built with mt genome sequences available in NCBI, which contained all protein and ribosomal RNA (rRNA) of previously sequenced plant mitochondrial genomes. Protein coding genes and ribosomal RNA genes were identified by performing local blast searches against the database. The tRNAscan-SE [33] was used to predict the tRNA genes. NCBI blast and local blast was used to identify putatively conserved regions among different plant mt genomes. The gene map was created by OGDraw (http://ogdraw.mpimp-golm.mpg.de/).

AB-blast was used to identify repeat sequences in *G. hirsutum* and other plant mt genomes (Table S5) as described previously [4], [9], [13]–[14], [18]–[19], [38]–[45]. The repeat sequence distribution map was drawn by Circos. The genome was searched against itself and local Perl scripts were adopted to run detail analysis. We used local R scripts to identify gene clusters by comparing every two mt genomes. Then we used the MEGA 5.0 to draw phylogenetic tree based on clustered genes. These 21 genes were 17 respiratory complex genes (*atp1*, *atp4*, *atp6*, *atp8*, *atp9*, *cob*, *cox1*, *cox3*, *nad1*, *nad2*, *nad3*, *nad4*, *nad4L*, *nad5*, *nad6*, *nad7*, *nad9*) and four cytochrome c biogenesis genes (*ccmB*, *ccmC*, *ccmFC*, *ccmFN*) (Table S4).

## Supporting Information

Figure S1 Phylogenetic trees of NADH dehydrogenase genes and cytochrome c biogenesis genes. The ML tree (A) and NJ tree (B) were based on NADH dehydrogenase genes. The ML tree (C) and the NJ tree (D) were based on cytochrome c biogenesis genes.
(TIF)

Figure S2 Phylogenetic trees of ATPase genes. The ML tree (A) and the NJ tree (B).
(TIF)

Figure S3 Phylogenetic trees of apocytochrome b genes and cytochrome c oxidase genes. The ML tree (A) and NJ tree (B) were based on apocytochrome b genes. The ML tree (C) and the NJ tree (D) were based on cytochrome c oxidase genes.
(TIF)

Table S1 Partial primers of PCR in genome assembling.
(DOC)

Table S2 Genes annotated in the *Gossypium hirsutum* mt genome.
(DOC)

Table S3 Size of shared sequences among 25 plant mt genomes.
(XLS)

Table S4 Information of genes in phylogenetic tree.
(DOC)

Table S5 Information of mitochondrial genomes involved in this study.
(DOC)
