# Supplementary figures and images for: The Complete Mitochondrial Genome of Gossypium hirsutum and Evolutionary Analysis of Higher Plant Mitochondrial Genomes
Source: PLoS One. 2013 Aug 5;8(8):e69476. doi: 10.1371/journal.pone.0069476 (PMC3734230; doi:10.1371/journal.pone.0069476)

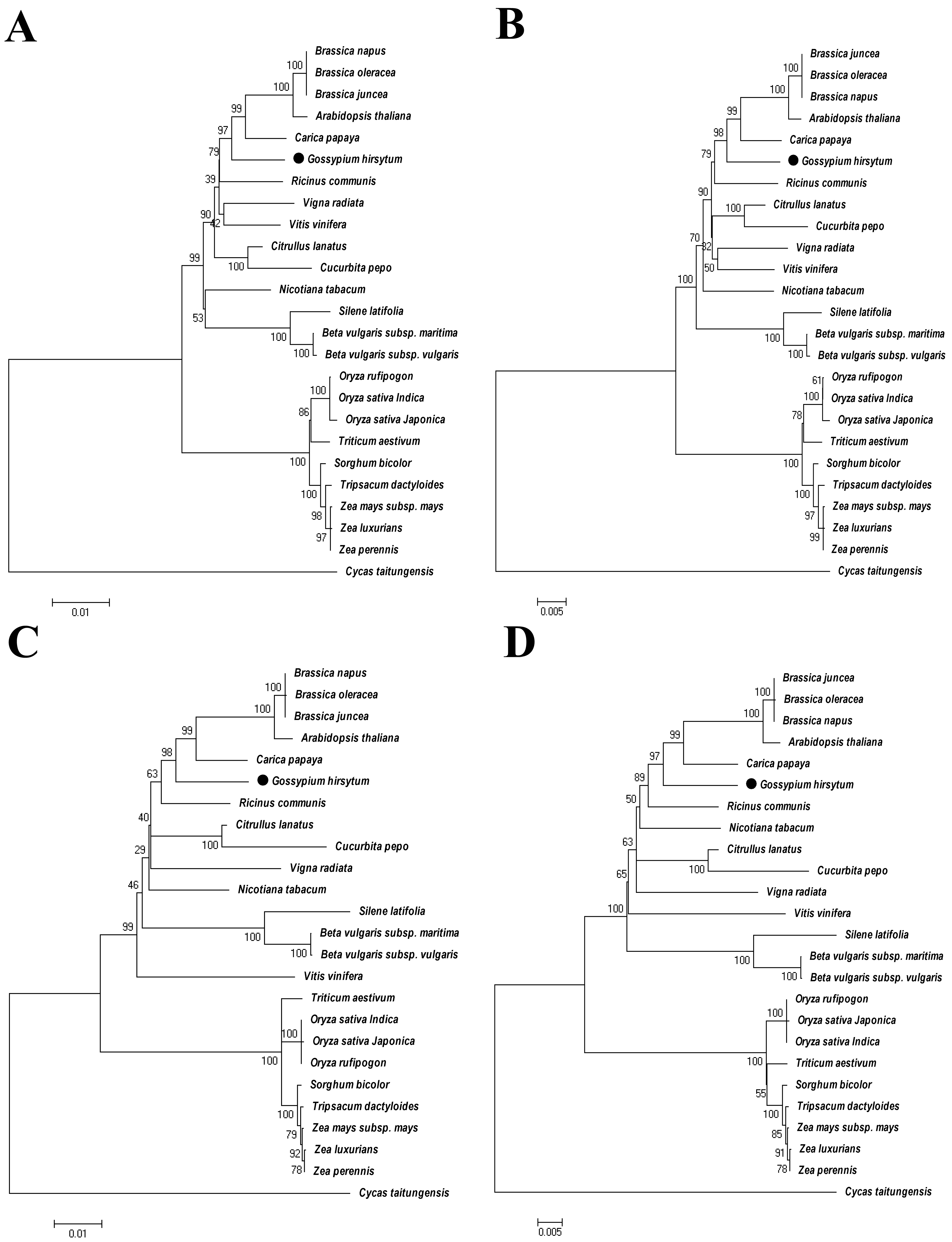

Supplement: Figure S1 — Phylogenetic trees of NADH dehydrogenase genes and cytochrome c biogenesis genes. The ML tree (A) and NJ tree (B) were based on NADH dehydrogenase genes. The ML tree (C) and the NJ tree (D) were based on cytochrome c biogenesis genes. (TIF) [file pone.0069476.s001.tif]

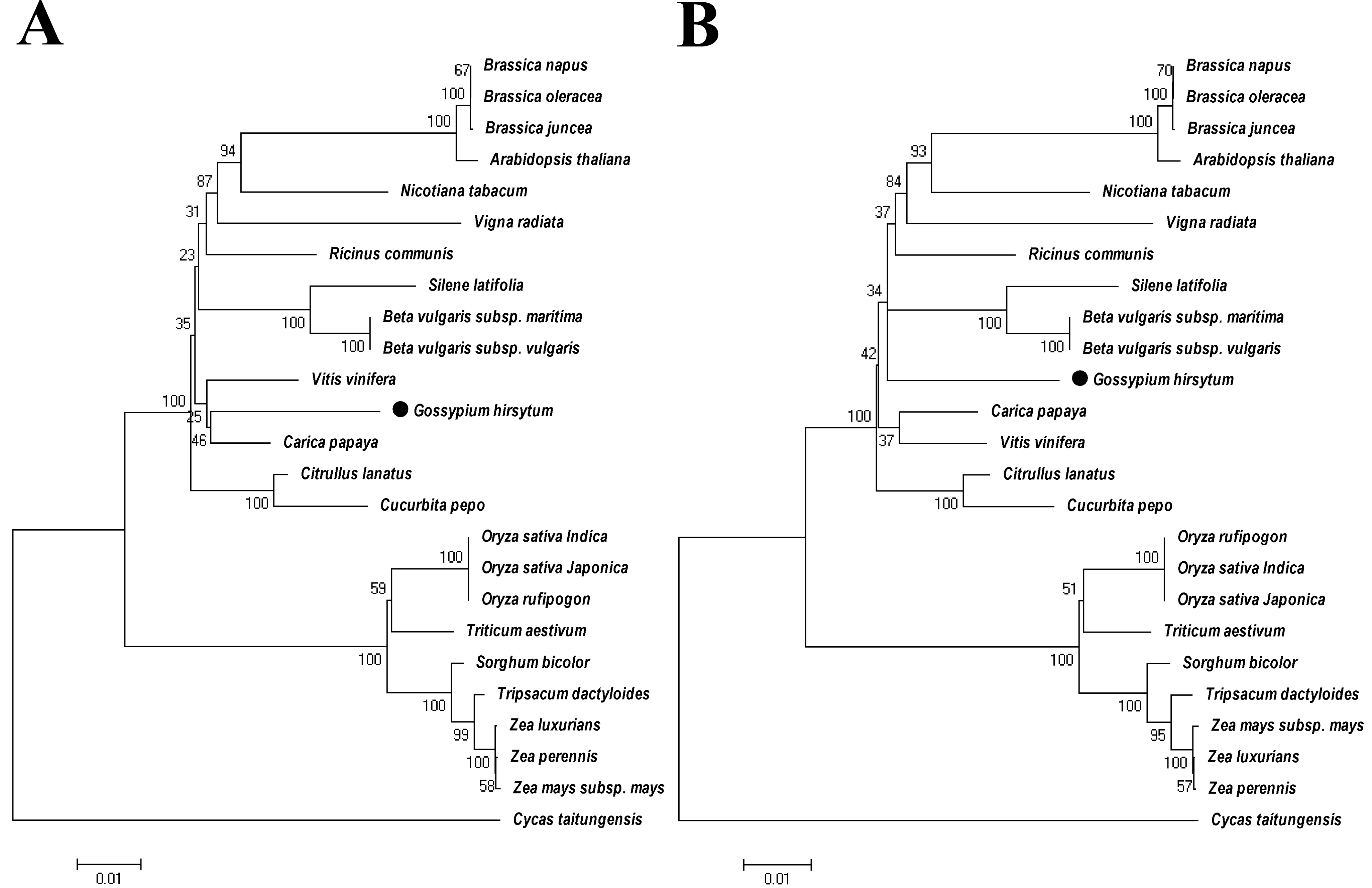

Supplement: Figure S2 — Phylogenetic trees of ATPase genes. The ML tree (A) and the NJ tree (B). (TIF) [file pone.0069476.s002.tif]

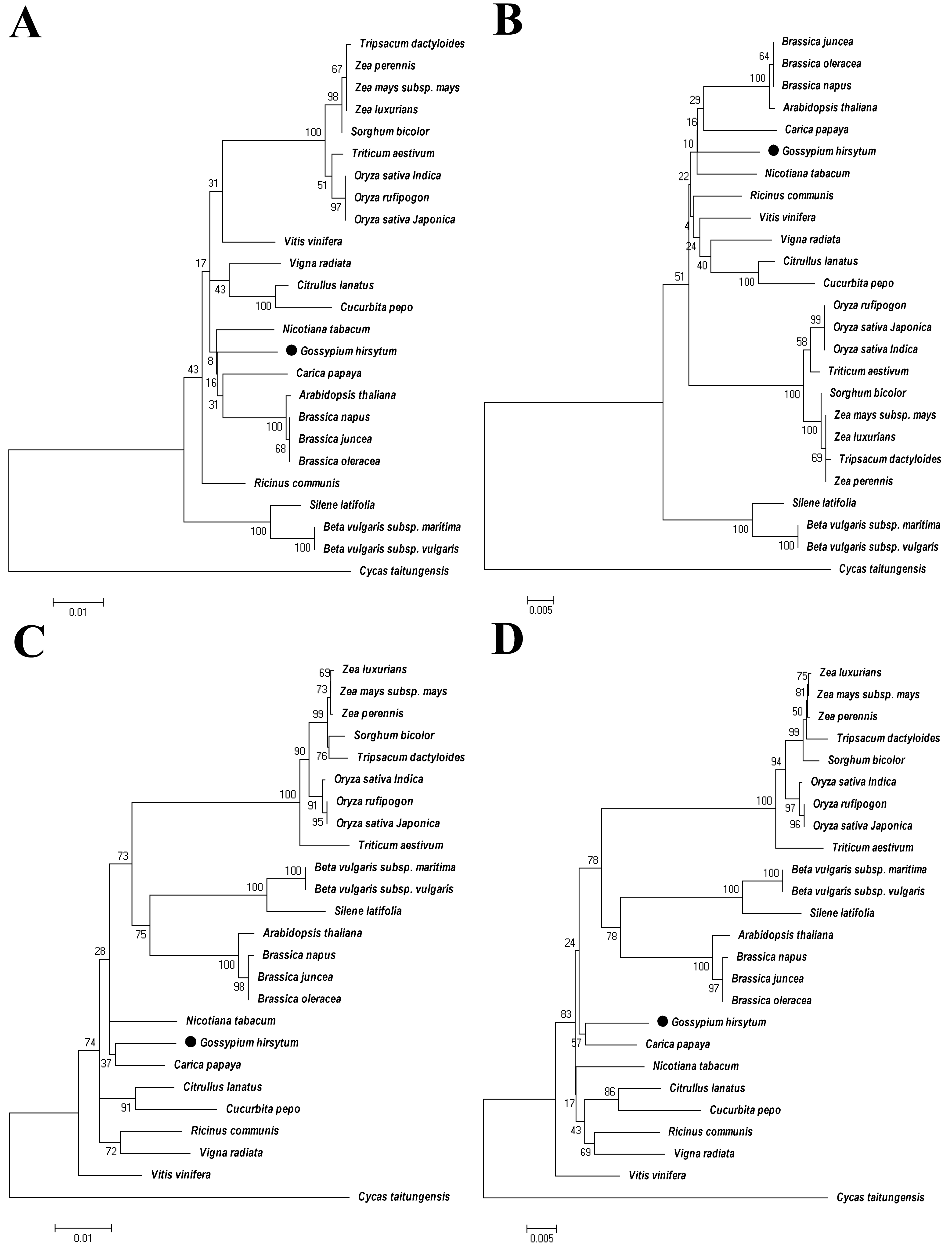

Supplement: Figure S3 — Phylogenetic trees of apocytochrome b genes and cytochrome c oxidase genes. The ML tree (A) and NJ tree (B) were based on apocytochrome b genes. The ML tree (C) and the NJ tree (D) were based on cytochrome c oxidase genes. (TIF) [file pone.0069476.s003.tif]
