# Supplementary material for: The Complete Mitochondrial Genome of Gossypium hirsutum and Evolutionary Analysis of Higher Plant Mitochondrial Genomes
Source: PLoS One. 2013 Aug 5;8(8):e69476. doi: 10.1371/journal.pone.0069476 (PMC3734230; doi:10.1371/journal.pone.0069476)
Supplement: Table S1 — Partial primers of PCR in genome assembling. (DOC) [file pone.0069476.s004.doc]

**Table S1**. Partial primers of PCR in genome assembling.

| Primers | | | | Sequence | | Primers | | | | Sequence | |
| --- | --- | --- | --- | --- | --- | --- | --- | --- | --- | --- | --- |
| P1 | | R | | CTATTGATGTAGCCTCTTGTCGC | | P42 | | R | | AGAATGCCAAACCAAAGACGAAG | |
|  | F | | CGCTCACTTCTACTCCTGTCCTG | |  | | F | | GCTGAACGACTGAAAGCCCAAC | |  |
| P2 | | R | | TGCTGAGATACTGGATGCTTACCG | | P43 | | R | | GGGGGACGGGGAAGCAAAGC | |
|  | F | | CAGTTGGTTGGATAAAGGTTGGAG | |  | | F | | GGGCCTTCCACTGCGGGAAC | |  |
| P3 | | R | | AAGGACGGGTCACCTGCCGA | | P44 | | R | | AGCCCGCCTAGCCTACGAGG | |
|  | F | | TCTCTCGCCCAGCTTTCGCC | |  | | F | | ACGCCCCTCTCTTCGGGACC | |  |
| P4 | | R | | GAGATTCCTTGATGCCGTTCGC | | P45 | | R | | CCAAGCCGCACGGAAAGGGT | |
|  | F | | GAGATGTTCCATTCTTCCCACG | |  | | F | | TCCGCCCTGGTTAGCTCGGT | |  |
| P5 | | R | | GGATGGATTTAGGAGTCTTTGTGC | | P46 | | R | | GCAGTCAGCCGCTTCCCCTG | |
|  | F | | GCTGTTCCCGTAACTCCACTCTC | |  | | F | | CGAGCGAGAGCCCAAACGGG | |  |
| P6 | | R | | TCATGTGGCCGAGCGAAGCG | | P47 | | R | | GCAGCACGCTGGCACTGACT | |
|  | F | | CCGGTTGCTCCTGCTGCTCC | |  | | F | | AGGCCCACACCCCTCCCATT | |  |
| P7 | | R | | CACCTGGACAGAAAGACCCTATG | | P48 | | R | | GCGGCGGGAGTCAACCATCC | |
|  | F | | CTGCTACGCTTCCTCCTTGTCG | |  | | F | | GCCACCCAGGGTTGGAACCC | |  |
| P8 | | R | | CCCCCGGTCACCCAAGGAAT | | P49 | | R | | ACAGGGCCCCATCACGCTCT | |
|  | F | | TGGCGTCGGAGGAACTCCGT | |  | | F | | AGGGGTCCATCAAGGGAGGGA | |  |
| P9 | | R | | CGGTCCCCGGTAGCCCAAGA | | P50 | | R | | TACCGCTCCGTGGGGCTACC | |
|  | F | | CCTGGGGCTGCAACTGAGGG | |  | | F | | CCATGGTTGCCGCCGGCTAA | |  |
| P10 | | R | | CCCGCCTATTGGCGTGTCTGG | | P51 | | R | | TGATCCCGCCCCACAGGAGG | |
|  | F | | AAGCCCCTCCTGTTGGCGTG | |  | | F | | CGCCCGGCTGGCATTCTTGA | |  |
| P11 | | R | | CCTCCCTTGCCTGCCAGCAC | | P52 | | R | | AGCAAGCGCTACGCCCCTTT | |
|  | F | | ATGCCCCTTTCCCACGCCAC | |  | | F | | ATAGCGTTGCACTCGGGGCG | |  |
| P12 | | R | | GGACGGAGCCGTATGACGCG | | P53 | | R | | TCGGGCTGGCCCTTGCCTAT | |
|  | F | | TAGTCGACCTCCGGCCTGCC | |  | | F | | GGACCTGAACCGTGGTGGTGC | |  |
| P13 | | R | | ACTGTGGAGCACACCGGAGC | | P54 | | R | | GACGCTGGGTCGGGGTTTGG | |
|  | F | | TAGGATTCCCCCGCACCCCC | |  | | F | | CCTTGCTTCTGCTCGCCGCT | |  |
| P14 | | R | | CGAGGTCCCTTGTTCTCTGGTC | | P55 | | R | | GCAATGGGATGGATGGTAGAGG | |
|  | F | | GTACTCTTACGCTCCTTCCTCTCC | |  | | F | | CGCATTTATGATTCCAGCCGAG | |  |
| P15 | | R | | CATTCGCTCACTTACGCTGTCC | | P56 | | R | | GCGAGCACCTATTACCCCACTAC | |
|  | F | | CGTAGAAGGAGATAACAGGTGGAGC | |  | | F | | CTTGGAGTGGGAGTGTAGCGTAGG | |  |
| P16 | | R | | AGCAAGTGTGCCGGGGATGC | | P57 | | R | | AAGCAGTCTGTCGGTCTAAGTCC | |
|  | F | | CTTGCTGGGGATCGGCGTGG | |  | | F | | GATTTCGTGGGTTATCATAGAGG | |  |
| P17 | | R | | CCACGCCGATCCCCAGCAAG | | P58 | | R | | GCCCCCGTCTTTCACTGGCC | |
|  | F | | GCTTCGTGCCGCACTGGAGT | |  | | F | | TGACCGCTGCCCCCTCGTTA | |  |
| P18 | | R | | CTGAACGACTGAAAGCCCAACC | | P59 | | R | | GCCATACCATACTATCCATACAGG | |
|  | F | | CTCGTGTCGTGTAGTGTAGCAAG | |  | | F | | CGTTGTTGGTCCTTCTTTCAC | |  |
| P19 | | R | | CAACTGTGGGTAAGGACGAAGG | | P60 | | R | | TGCTTACGCGATGGTGTGCGT | |
|  | F | | AACCGAAGTAATGGAAGTTTGTGG | |  | | F | | CCTAGTGCCGTGAAAGGGCGG | |  |
| P20 | | R | | TGTGCCCCATGGAAAGGGCG | | P61 | | R | | TCTTGAGCCCCGTGGCAAGC | |
|  | F | | CCGATTCGCCTTGACCCGGA | |  | | F | | TCCGTCACAGAAGCCCGGGA | |  |
| P21 | | R | | GTCCGAGTGGCGCAATGGCT | | P62 | | R | | ACGGTGGCAGCTGCGGAATC | |
|  | F | | GCTGCGCTCCTGCTGTCCAA | |  | | F | | CATGCTGGCACCTTGGCGGA | |  |
| P22 | | R | | TGGTGCTTCCCCAGCCACCT | | P63 | | R | | CCATCGCGCTGTCCTTCCCG | |
|  | F | | AGGCACCAACCGGGAACCCT | |  | | F | | CCCCCTACTGAGCCGTGGGG | |  |
| P23 | | R | | GGCGGGCGAAGGGTCAACTC | | P64 | | R | | CTTCTCGGCTTCACTATGTTCG | |
|  | F | | TACCGACAGCAGCTCCCGCT | |  | | F | | GCTTTACTTCCTCGCCTTAGATTC | |  |
| P24 | | R | | CATCTCGCGCCTATGGGCCG | | P65 | | R | | CCAGTCCTTGTCGCAGCGCA | |
|  | F | | TCAACGCTTCCAACGCCCGT | |  | | F | | GCAAGCAAGCGAACGGAGCG | |  |
| P25 | | R | | ACCGGGGCCTTTGACCCTCA | | P66 | | R | | TCCGAGTCGTCCAGCTGGCA | |
|  | F | | CGGCTGATACTCCCGCGCAG | |  | | F | | CTCTGCAGGGCAGCGCCATT | |  |
| P26 | | R | | AAGCCCTCGCCCGACAGTCT | | P67 | | R | | TGAGCGCGAGGGGAAGGGTT | |
|  | F | | GGGCGCTTCGTGGCCCTATC | |  | | F | | TGCTTCGACCGATGGGCTGC | |  |
| P27 | | R | | GAACCCAACTCGTCGGACTCAC | | P68 | | R | | GCGTTCATCCCCATCTCTTGTCC | |
|  | F | | GCTTAGTCAGACCAACCGTATCG | |  | | F | | GAACCTACCACGACCACCATCCG | |  |
| P28 | | R | | GGACCCGACCCGGTCACAAG | | P69 | | R | | GCTGGGGTTCGTGGTCGTGG | |
|  | F | | GGCGCATGAGAGGCCAGCAG | |  | | F | | GATGGTTGCGCAGGGCCCTT | |  |
| P29 | | R | | ACAACGGCCCAAGCGACCAG | | P70 | | R | | CCGGCCTACTACGGGCACCT | |
|  | F | | CGTTCCCCGACTGGGGAGGT | |  | | F | | AAGCCGCCCTACCTCCGGAG | |  |
| P30 | | R | | GCTTTGCGATACGGACGAACAC | | P71 | | R | | ACGACGGTGGAAGTAGAGTGGC | |
|  | F | | AGTCCTCTCACTACCAACCAGCA | |  | | F | | CCTTGATAGCCTTGAGTTGTTGG | |  |
| P31 | | R | | TATGAAGGAACGAAACGGTGG | | P72 | | R | | TAATCCGATGCCCACCCCGC | |
|  | F | | GAGCGGAGCACAAGCGAACC | |  | | F | | ACCACGAGATGTGGAGCGCG | |  |
| P32 | | R | | ATGCGTGGACCTGGAATGAC | | P73 | | R | | GGGCACGAACGGGAGCCTTT | |
|  | F | | CTGTGATAGTTCCGAATGTTGATG | |  | | F | | CTTACCGCTCGCTCCGTGGG | |  |
| P33 | | R | | TGAGTGTTCGCCCTTGGTCTACGC | | P74 | | R | | TTCTGCTGGTGCGCAGGAGC | |
|  | F | | GCTATTCCTTTCACACCGCTCGC | |  | | F | | CCGCCCCATGGCCTAGCCTA | |  |
| P34 | | R | | CGTAACCTCACCCTTTCCATCG | | P75 | | R | | GGCGACTACGACCCACCCCT | |
|  | F | | CAAGACAGGAAGAACGCAACGC | |  | | F | | GGAATCCCCCGGCCTGTGCT | |  |
| P35 | | R | | CTACGTGCATAGGGCGCCGG | | P76 | | R | | GGGGCTTTGGCACGTCGAGG | |
|  | F | | CGTCCTTCCCCCTGCCTCGA | |  | | F | | AGAGATGCGCCTTTCGCCGG | |  |
| P36 | | R | | AGGGCGGTGCTCTGACCGAT | | P77 | | R | | ACTGCAAACCTGCACCTACCCC | |
|  | F | | AGTAGCCCTCGAGGAGTCCGT | |  | | F | | AGGGAGGGAGCACTCGTGGC | |  |
| P37 | | R | | CTCTATCAGGCGGCGGCAGC | | P78 | | R | | AGTACCCCCAGGCGTTCGCT | |
|  | F | | GCACAAGGCCCCTGCAGAGG | |  | | F | | AGGCAGGGCGCGTTAAGCAG | |  |
| P38 | | R | | GCACCTACGGCAGGATCCGT | | P79 | | R | | GGCTGCAGGCGGATGCTAGG | |
|  | F | | GCGCTTGCCCGCTCTACGTA | |  | | F | | GAAGCGCAGCCCCTCCATCG | |  |
| P39 | | R | | GCTCTGCAGGTGGGCACCAC | | P80 | | R | | CCCTGGGGCCCTTAGAGCGT | |
|  | F | | CCGAAGGCTCCGCAACACGT | |  | | F | | GCCGGGTCCTTGCTTCGCTT | |  |
| P40 | | R | | TCATGTGGCCGAGCGAAGCG | | P81 | | R | | CCATCGCGCTGTCCTTCCCG | |
|  | F | | CCGGTTGCTCCTGCTGCTCC | |  | | F | | CCCCCTACTGAGCCGTGGGG | |  |
| P41 | | R | | AGCTCAGCTTTGCGGAGCGG | |  | |  | |  | |
|  | F | | AGCTAGTCGACGAGGGGGCG | |  | |  | |  | |  |

F forward; R reverse.
