# Supplementary material for: The Complete Mitochondrial Genome of Gossypium hirsutum and Evolutionary Analysis of Higher Plant Mitochondrial Genomes
Source: PLoS One. 2013 Aug 5;8(8):e69476. doi: 10.1371/journal.pone.0069476 (PMC3734230; doi:10.1371/journal.pone.0069476)
Supplement: Table S2 — Genes annotated in the Gossypium hirsutum mt genome. (DOC) [file pone.0069476.s005.doc]

**Table S2. Genes annotated in the *Gossypium hirsutum*** mt genome

| Type | Gene | Location |  | Type | Gene | Location |
| --- | --- | --- | --- | --- | --- | --- |
| **Functional proteins** |  |  |  |  |  |  |
| Complex I | *nad1-1*^† ‡^ | 211674..212060 |  | Complex V | *atp1* | **276616..278139** |
|  |  | **453235..454473** |  |  | *atp4* | 430861..431445 |
|  |  | **81155..84907** |  |  | *atp6* | **494665..495480** |
|  | *nad1-2*^†^ | **610630..611868** |  |  | *atp8* | 224444..224908 |
|  | *nad2*^† ‡^ | **418598..420365** |  |  | *atp9* | 162829..163140 |
|  |  | 38617..43354 |  | Cytochrome c biogenesis | *ccmB* | 95019..95639 |
|  | *nad3* | **129803..130159** |  |  | *ccmC* | 340798..341550 |
|  | *nad4*^†^ | **314159..322726** |  |  | *ccmFC*^†^ | 47731..50039 |
|  | *nad4L* | **467216..467518** |  |  | *ccmFN* | 507206..508942 |
|  | *nad5*^† ‡^ | 163361..165659 |  | Ribosomal proteins (LSU) | *rpl2* | **532722..533726** |
|  |  | **535923..535944** |  |  | *rpl5* | **534224..534805** |
|  |  | **401063..401212** |  |  | *rpl10* | **190279..190767** |
|  | *nad6* | 378914..379534 |  |  | *rpl16* | **593447..593881** |
|  | *nad7*^†^ | **115581..122114** |  | Ribosomal proteins (SSU) | *rps3-1*^†^ | **593853..597250** |
|  | *nad9* | **309687..310259** |  |  | *rps3-2*^† §^ | **437002..439858** |
| Complex II | *sdh3* | **421265..421699** |  |  | *rps4* | 565574..566671 |
|  | *sdh4* | **258268..258666** |  |  | *rps7* | **689..1135** |
| complex III | *cob* | **547956..549134** |  |  | *rps10*^†^ | **262587..263768** |
| Complex IV | *cox1* | **260808..262400** |  |  | *rps12* | **129383..129754** |
|  | *cox2*^†^ | 578221..580504 |  |  | *rps14* | **550498..550800** |
|  | *cox3* | **258594..259391** |  | Other genes | *matR* | **82219..84186** |
|  |  |  |  |  | *mttB* | **308702..309502** |
| **Ribosomal RNAs** |  |  |  |  |  |  |
|  | *rrn5* | **279256..279374** |  |  | *rrn26* | 67641..71014 |
|  | *rrn18* | **279538..281494** |  |  | *rrn26* | **250832..254207** |
| **Transfer RNAs** |  |  |  |  |  |  |
|  | *trnC* | 34837..34907 |  |  | *trnM* | **250397..250469** |
|  | *trnD* | 206335..206408 |  |  | *trnM* | **351190..351262** |
|  | *trnD* | **326179..326252** |  |  | *trnP* | 186050..186128 |
|  | *trnE* | 8005..8076 |  |  | *trnP* | **455837..455911** |
|  | *trnF* | **456132..456205** |  |  | *trnP* | **613232..613306** |
|  | *trnF* | **613527..613600** |  |  | *trnQ* | 359139..359210 |
|  | *trnG* | 356875..356946 |  |  | *trnS* | **91658..91744** |
|  | *trnN* | 37045..37116 |  |  | *trnS* | **456553..456640** |
|  | *trnY* | 38012..38094 |  |  | *trnS* | 488481..488567 |
|  | *trnH* | **5922..5995** |  |  | *trnS* | **613948..614035** |
|  | *trnI* | 341537..341622 |  |  | *trnV* | 195501..195572 |
|  | *trnK* | **92580..92652** |  |  | *trnW* | 186193..186266 |
|  | *trnM* | 53510..53590 |  |  | *trnW* | 501138..501211 |
|  | *trnM* | 67209..67279 |  |  | *trnSup* | 325893..325981 |
|  | *trnM* | 161960..162032 |  |  |  |  |

^†^ genes contain introns;

^‡^ genes that need trans-splicing;

^§^pseudo-gene

Boldface: transcription occurs on the antisense strand.
