# Supplementary material for: The Complete Mitochondrial Genome of Gossypium hirsutum and Evolutionary Analysis of Higher Plant Mitochondrial Genomes
Source: PLoS One. 2013 Aug 5;8(8):e69476. doi: 10.1371/journal.pone.0069476 (PMC3734230; doi:10.1371/journal.pone.0069476)
Supplement: Table S4 — Information of genes in phylogenetic tree. (DOC) [file pone.0069476.s007.doc]

**Table S4. Information of genes in phylogenetic tree**

| Gene | Protein | Complex |
| --- | --- | --- |
| *nad1* | NADH dehydrogenase subunit 1 | Complex I |
| *nad2* | NADH dehydrogenase subunit 2 |  |
| *nad3* | NADH dehydrogenase subunit 3 |  |
| *nad4* | NADH dehydrogenase subunit 4 |  |
| *nad4L* | NADH dehydrogenase subunit 4L |  |
| *nad5* | NADH dehydrogenase subunit 5 |  |
| *nad6* | NADH dehydrogenase subunit 6 |  |
| *nad7* | NADH dehydrogenase subunit 7 |  |
| *nad9* | NADH dehydrogenase subunit 9 |  |
| *cob* | apocytochrome b | Complex III |
| *cox1* | cytochrome c oxidase subunit 1 | Complex IV |
| *cox3* | cytochrome c oxidase subunit 3 |  |
| *atp1* | ATPase subunit 1 | Complex V |
| *atp4* | ATPase subunit 4 |  |
| *atp6* | ATPase subunit 6 |  |
| *atp8* | ATPase subunit 8 |  |
| *atp9* | ATPase subunit 9 |  |
| *ccmB* | cytochrome c biogenesis B | Cytochrome c biogenesis |
| *ccmc* | cytochrome c biogenesis C |  |
| *ccmFC* | cytochrome c biogenesis FC |  |
| *ccmFN* | cytochrome c biogenesis FN |  |
