# Supplementary material for: The Complete Mitochondrial Genome of Gossypium hirsutum and Evolutionary Analysis of Higher Plant Mitochondrial Genomes
Source: PLoS One. 2013 Aug 5;8(8):e69476. doi: 10.1371/journal.pone.0069476 (PMC3734230; doi:10.1371/journal.pone.0069476)
Supplement: Table S5 — Information of mitochondrial genomes involved in this study. (DOC) [file pone.0069476.s008.doc]

**Table S5. Information of mitochondrial genomes involved in this study.**

| Classification | | Taxa | Accession number | Reference |
| --- | --- | --- | --- | --- |
| Gymnosperms |  | *Cycas taitungensis* | NC_010303 | Chaw et al., 2008 |
| Angiosperms | Monocots | *Oryza rufipogon* | NC_013816 | Fujii et al., 2010 |
|  |  | *Oryza sativa subsp indica* | NC_007886 | Tian et al., 2006 |
|  |  | *Oryza sativa subsp japonica* | NC_011033 | Notsu et al., 2002 |
|  |  | *Sorghum bicolor* | NC_008360 |  |
|  |  | *Tripsacum dactyloides* | NC_008362 | Allen et al., 2007 |
|  |  | *Triticum aestivum* | NC_007579 | Ogihara, 2005 |
|  |  | *Zea luxurians* | NC_008333 | Allen et al., 2007 |
|  |  | *Zea perennis* | NC_008331 | Allen et al., 2007 |
|  |  | *Zea mays subsp. mays* | NC_007982 | Clifton et al., 2004 |
|  | Dicots | *Arabidopsis thaliana* | NC_001284 | Unseld et al., 1997 |
|  |  | *Beta vulgaris subsp maritima* | NC_015099 |  |
|  |  | *Beta vulgaris subsp vulgaris* | NC_002511 | Kubo et al., 2000 |
|  |  | *Brassica juncea* | NC_016123 |  |
|  |  | *Brassica napus* | NC_008285 | Handa, 2003 |
|  |  | *Brassica oleracea* | NC_016118 |  |
|  |  | *Carica papaya* | NC_012116 |  |
|  |  | *Citrullus lanatus* | NC_014043 | Alverson et al., 2010 |
|  |  | *Cucurbita pepo* | NC_014050 | Alverson et al., 2010 |
|  |  | *Gossypium hirsutum* | JX065074 | Current article |
|  |  | *Nicotiana tabacum* | NC_006581 | Sugiyama et al., 2004 |
|  |  | *Ricinus communis* | NC_015141 | Rivarola et al., 2011 |
|  |  | *Silene latifolia* | NC_014487 | Sloan et al., 2010 |
|  |  | *Vigna radiata* | NC_015121 | Alverson et al., 2011 |
|  |  | *Vitis vinifera* | NC_012119 | Goremykin et al., 2009 |
